# Physicochemical modelling of the retention mechanism of temperature-responsive polymeric columns for HPLC through machine learning algorithms

**DOI:** 10.1186/s13321-024-00873-6

**Published:** 2024-06-21

**Authors:** Elena Bandini, Rodrigo Castellano Ontiveros, Ardiana Kajtazi, Hamed Eghbali, Frédéric Lynen

**Affiliations:** 1 Separation Science Group, Department of Organic and Macromolecular Chemistry, Univeristy of Ghent, Krijgslaan 281 S4bis, Ghent, 9000 Belgium; 2 School of Electrical Engineering and Computer Science, KTH Royal Institute of Technology, Stockholm, 11428 Sweden; 3 Packaging and Specialty Plastics R&D, Dow Benelux B.V., Terneuzen, 4530 AA the Netherlands

**Keywords:** Retention mechanism, Machine learning, Molecular descriptors, Temperature-responsive liquid chromatography

## Abstract

Temperature-responsive liquid chromatography (TRLC) offers a promising alternative to reversed-phase liquid chromatography (RPLC) for environmentally friendly analytical techniques by utilizing pure water as a mobile phase, eliminating the need for harmful organic solvents. TRLC columns, packed with temperature-responsive polymers coupled to silica particles, exhibit a unique retention mechanism influenced by temperature-induced polymer hydration. An investigation of the physicochemical parameters driving separation at high and low temperatures is crucial for better column manufacturing and selectivity control. Assessment of predictability using a dataset of 139 molecules analyzed at different temperatures elucidated the molecular descriptors (MDs) relevant to retention mechanisms. Linear regression, support vector regression (SVR), and tree-based ensemble models were evaluated, with no standout performer. The precision, accuracy, and robustness of models were validated through metrics, such as *r* and mean absolute error (MAE), and statistical analysis. At $45\,^{\circ }\hbox {C}$, logP predominantly influenced retention, akin to reversed-phase columns, while at $5^{\circ }\hbox {C}$, complex interactions with lipophilic and negative MDs, along with specific functional groups, dictated retention. These findings provide deeper insights into TRLC mechanisms, facilitating method development and maximizing column potential.

**Supplementary Information:**

The online version contains supplementary material available at 10.1186/s13321-024-00873-6.

## Introduction

**Fig. 1:**
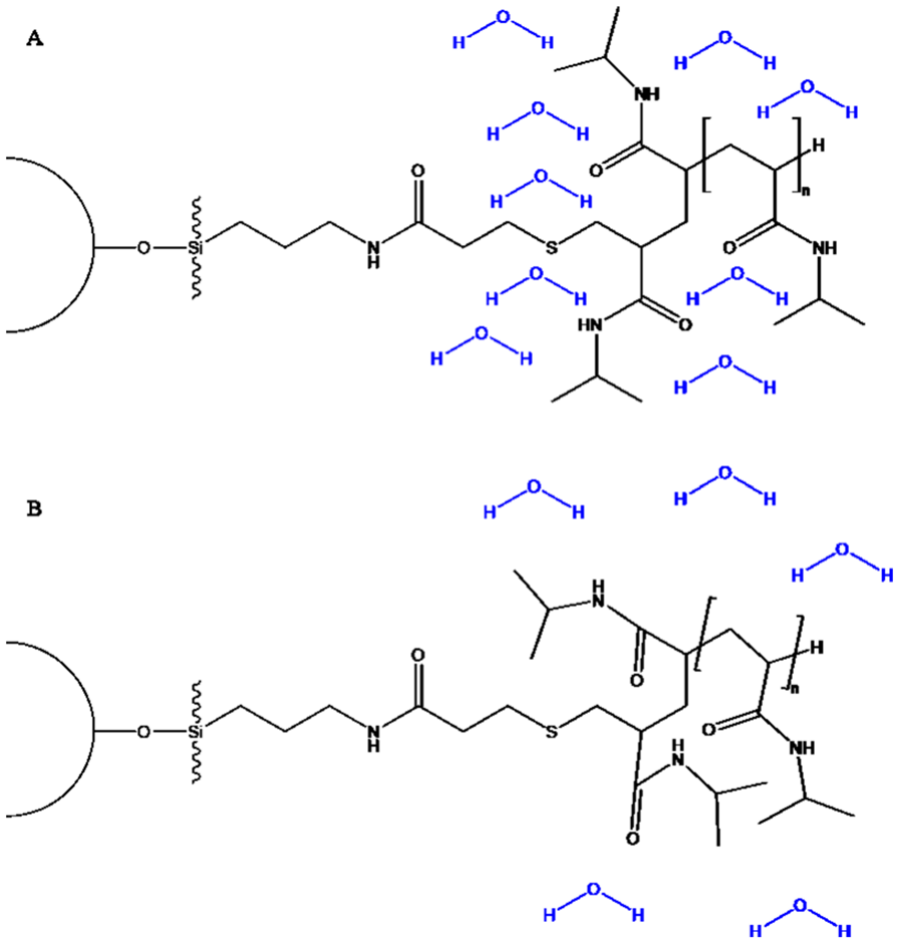
TRLC stationary phase structure in the hydrated form or coil conformation, below the LCST (**A**) and in the dehydrated form or globule conformation, above the LCST (**B**)

Temperature-responsive liquid chromatography (TRLC) is an emerging mode in HPLC (high-performance liquid chromatography), that can be considered a greener alternative to reversed-phase separation, as it works in purely aqueous conditions. The retention inside the column is therein controlled by the temperature instead of the solvent composition [1]. TRLC columns are packed with temperature-responsive polymers attached to silica particles. The change in conformation of the polymers with temperature allows for the change in retention. Polymers used in chromatography are the ones with a lower critical solution temperature (LCST) behaviour, meaning that they are present in solution in their solvated form at low temperatures and de-solvate at high temperatures. The temperature at which this change happens is defined as LCST, ideally, for chromatography applications, this should be in an acceptable range to not affect water viscosity, hydrothermal stability of the silica, and analyte degradation. For these reasons polymers with LCST between 0 and $45\,^{\circ }\hbox {C}$ are preferred. In this study, PNIPAAm (Poly-N-isopropyl acrylamide) was used inside the HPLC column due to its wide use, in many fields more than chromatography [2], making its synthesis and characteristics reliable in terms of repeatability, and because of its LCST of $32\,^{\circ }\hbox {C}$, suitable for HPLC applications [3]. Figure 1 shows the structure of the stationary phase of PNIPAAm packed columns below (A) and above (B) the polymer LCST. This technique has several advantages over traditional reversed-phase liquid chromatography (RPLC), including reduced solvent consumption and waste generation, as well as improved compatibility with mass spectrometry due to the use of a purely aqueous mobile phase. During the past years, different stimuli-responsive polymers have been studied as well as different conditions for their use [4]. Recently the use of TRLC with a small percentage of organic modifier in the mobile phase was also demonstrated as a possibility, broadening the number of molecules that can be analyzed [5]. The potential of TRLC has been exploited in many applications and used to solve numerous issues that would be present with other modes. It proved useful to use TRLC as a first dimension in comprehensive 2D-LC to overcome refocusing problems and increase sensitivity [6, 7]. It was used with a refractive index detector (RID) to perform temperature gradient elution as an alternative to solvent gradient which is not possible with RID [8]. Despite the ongoing research to broaden the use of the technique, so far, a detailed explanation of the retention mechanism has been lacking. As of today, there is awareness of some similarities with RPLC, especially at temperatures above the LCST, while at low temperatures the mechanism is more reminiscent of adsorption or normal phase LC. In general, more apolar molecules have higher retention, however, the retention is also increased for molecules with hydrophobic chains containing additional polar functions. The change in separation mechanism is gradual around the LCST, and it can be visualized through Van’t Hoff plots where the slope of the curve is negative for TRLC, and it presents a small step at the LCST [9]. In this work, the aim is to gain a deeper understanding of the separation mechanisms involved in TRLC with the perspective that this knowledge can help obtain easier and faster method development in future applications and better control over column manufacturing and, consequently, selectivity. The approach proposed starts with the construction of a prediction model for the retention factor (k) at two different temperatures (above and below the polymer PNIPAAm LCST, 45 and $5\,^{\circ }\hbox {C}$) followed by the elucidation of the most important features influencing the model. The retention factor (*k*) is a key parameter in chromatography, it is a measure of the relative distance that a component travelled inside the column. It depends on various factors such as the properties of the analyte, the mobile phase, and the stationary phase. Understanding how *k* varies with these factors can help optimize the separation conditions and improve the efficiency and accuracy of TRLC. One way to study the relationship between *k* and the properties of analytes is to use molecular descriptors (MDs), which are numerical values that represent different aspects of molecular structure and physicochemical properties [10]. They reflect the molecular features of a compound and help to establish the relationship with the chromatographic data [11]. Despite being sometimes redundant or highly correlated, they are essential to elucidate the complex interaction between the analytes and the stationary phase [12]. Currently, the number of MDs available is almost uncountable, the dataset used in this work, for example, provides 5666 possible descriptors for each molecule and they are sometimes very challenging to interpret and link to the retention of the molecule [13]. For this reason, the way they are pre-processed is of utter importance [14]. MDs can be divided into 4 classes: 0, 1, 2, 3, and 4-dimensional ones. The 0D are derived directly from the molecular formula, hence, they are independent of the structure (e.g., molecular weight, number of atoms). 1D descriptors consider the functional groups in the molecule, and 2D descriptors are the results of the topological representation of the molecule (bonds and interactions between atoms). Additionally, 3D descriptors are geometrical representations of the molecule, and 4D are derived from stereo electronic representation (such as the distribution of some properties in the molecule). A comparative analysis was conducted among various machine learning-based methodologies to forecast the parameter *k*. From these methodologies, the goal was to identify and prioritize the most relevant features. This process aimed to enhance the comprehension of TRLC, providing a more profound understanding of the underlying mechanisms governing the separation mode, specifically focusing on its behaviours at both high and low temperatures. Linear regression is used as a baseline model for being easy to interpret and having a low computational cost. However, it assumes a linear relationship between the variables, it is not able to capture more complex relations, and it is prone to overfitting when the input contains many variables [15]. For this latter reason, different types of regularization were implemented to improve unreliability [16]. Specifically, Lasso [17], Lasso LARS, Ridge [18], and Elastic Net [19] were considered. These regularization methods reduce overfitting by shrinking the coefficients of the model, the difference is in the way they impose the penalty on the coefficients. The selected machine learning models were four tree-based ensemble models: Random Forest (RF), Extra Trees Regressor (OXT), Gradient Boosting (GB) and Extreme Gradient Boosting (XGBoost), and one more model, Support Vector Regression (SVR). Random Forest and Extra Trees Regressor are bagging methods, which train multiple trees on random subsets of data and features and then aggregate their predictions [20, 21]. Random Forest is well known as one of the most accurate and fast learning models independent of the nature of the dataset, and it is usually used as a benchmark for non-linear models’ comparison [22]. Gradient Boosting and XGBoost are boosting methods, which train multiple trees sequentially and each tree tries to correct the errors made by the previous ones [23]. In general, boosting models tend to outperform others, XGBoost has been demonstrated to deliver more accurate predictions with many different datasets [22]. In retention time prediction these algorithms are the most used ones together with Artificial Neural Networks (ANNs) [24–30]. In this work, ANNs are not considered as they cannot provide feature importance with weight, and for the same reason, SVR is only tested with a linear kernel. The novelty of this work lies in its comprehensive approach to understanding the separation mechanisms in TRLC and its potential implications for method development and column manufacturing. While previous studies have explored TRLC, a detailed explanation of the retention mechanism has been lacking. This work aims to fill this gap by constructing a prediction model for the retention factor at different temperatures and identifying the key features influencing the model.

## Experimental

### Materials

Milli-Q grade water (18.2 m$\Omega$) was purified and deionized in-house by a Milli-Q plus instrument from Millipore (Bedford, USA). Formic acid (FA) was supplied by Acros (Geel, Belgium). The list of compounds includes 139 chemicals, which can be found in SI section S1, and it includes the values of *k* for 45 and $5\,^{\circ }\hbox {C}$. The compounds chosen are from different classes, to have a heterogeneous dataset. Many are chemicals of relevant interest to the environment such as pharmaceuticals, steroids, and pesticides. Most of the compounds present a logP value in a suitable range for RPLC. The chemical standards are prepared in 50:50 water/acetonitrile at a concentration of 0.1 mg $\hbox {mL}^{-1}$. PNIPAAm columns (50 $\times$ 4.6 mm, silica particles 5 $\upmu$m, 120 Å) were manufactured as described in Baert et al. [9], whereby the packing material was additionally end-capped with acetic anhydride (99$\%$, Acros) (for polymer characteristics see supporting information S2).

### Chromatographic data acquisition

The HPLC analyses were performed on an 1100 series HPLC system (Agilent Technologies, Waldbronn, Germany), made of an (1100) binary pump equipped with an (1100) degasser, an (1100) auto-injector, a (1100) variable wavelength detector (VWD) and a (1200) RID. The column temperature was controlled using a water/glycol bath (Julabo, Seelbach, Germany, model F10). Short 0.13 mm ID connection tubing was used between the devices and Viper (Thermo Fisher Scientific, Germany) connections were used for the connections to (950 x 0.1 mm) and from (750 x 0.1 mm) the column. Data collection was done with ChemStation (Rev. B.04.03 [16], Agilent). An identical method is used for both temperatures, 5 and $45\,^{\circ }\hbox {C}$, that considers the optimal conditions for the column to ensure elution in a reasonable time. The method is summarized in Table 1, the UV wavelength was selected depending on the analyte. A subset of compounds was also analyzed with a Thermo Fisher Q Exactive Orbitrap mass spectrometer (Thermo Fisher Scientific, Germany) for further confirmation of the peak. The final dataset is composed of 139 compounds with respective values of *k* at 5 and $45\,^{\circ }\hbox {C}$. The experimental dataset includes molecules from different classes, from steroids containing only C, H and O, to sulfonamides, nitrogen-containing molecules, and chlorinated and fluorinated ones, to have a heterogeneous dataset that makes it possible to understand the interactions with different types of analytes. At $45\,^{\circ }\hbox {C}$ the average column dead time is 0.96 min, and the *k* range is 0.14-53.55, while at $5\,^{\circ }\hbox {C}$ the average dead time is 0.93 min and the range of *k* is 0-32.77 (full dataset in S1).

**Table 1:** Analysis method conditions

Mobile phase	Water + 0.1% v/v FA
Flow rate	0.7 mL $\hbox {min}^{-1}$
Injection volume	5 $\upmu$L
Column temperature	5 and $45\,^{\circ }$C

### Molecular descriptors

The in silico obtained dataset is composed of 5666 molecular descriptors derived from OCHEM [31]. The MDs were computed from isomeric SMILES (simplified molecular-input line-entry system) notation of the chemical compounds [32]. The molecules were pre-processed with Corina. The optimization process involves standardization, neutralization, salts removal and clean structure. After that, the MDs were calculated through AlvaDesc v.2.0.14. The MDs with low variability, very stable throughout the dataset (standard deviation < 0.01) and high collinearity (Pearson correlation coefficient > 0.95) were removed since they contain very similar or not useful information. The final dataset consisted of 1654 MDs, and this was used to train the models. Descriptors value for the last input to the model were normalized by a min-max scaler in the range [0, 1], to give each variable the same weight (Eq. 1):

1 $$\begin{aligned} X _{scaled} = \frac{X - X _{min}}{X _{max} - X _{min}} \end{aligned}$$

Normalization of the input is advised as it improves performance and numerical stability and prevents features with large value ranges from dominating over other features during the training [33].

### Prediction models

**Fig. 2:**
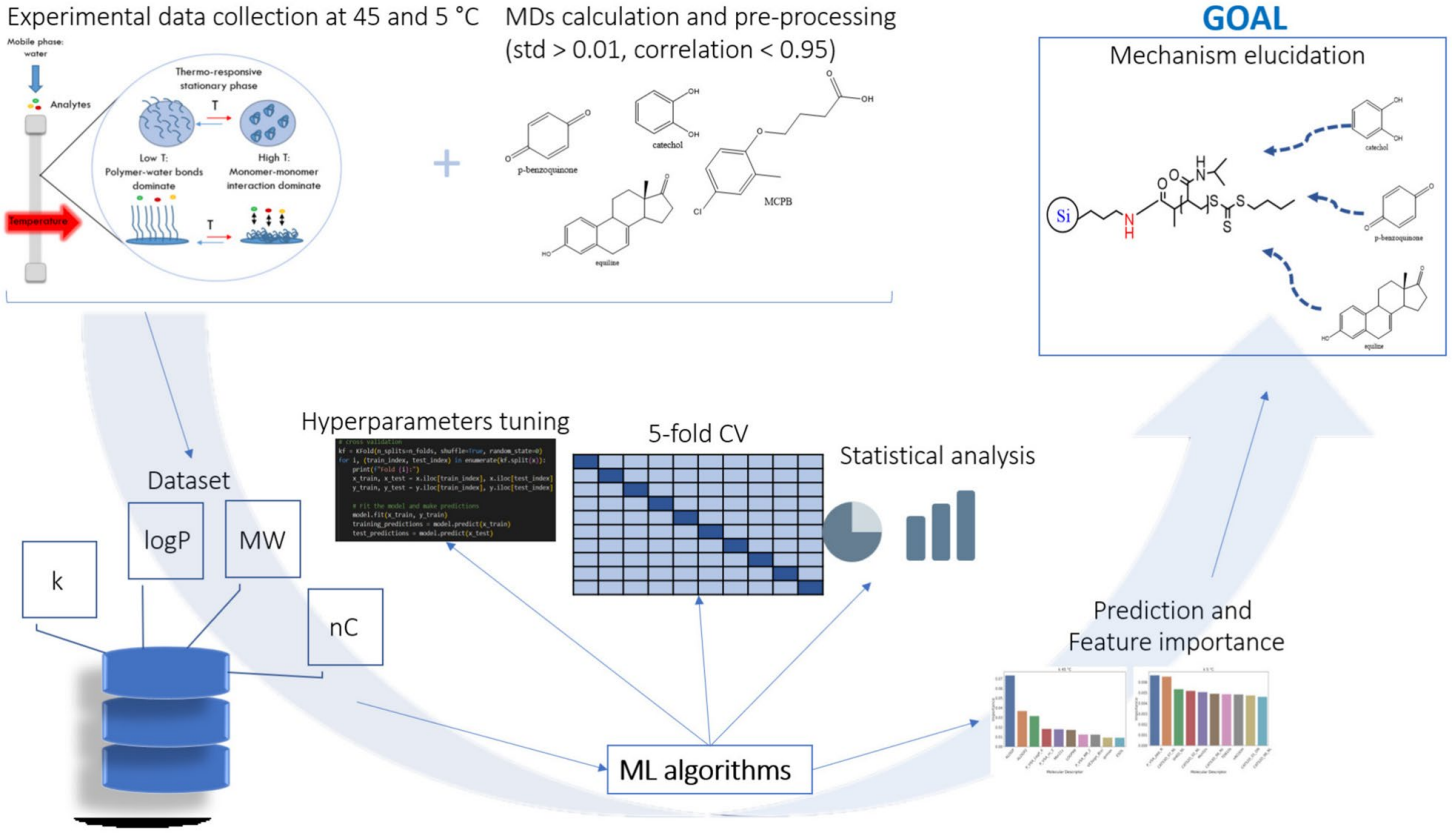
Workflow for the modelling of the retention mechanism of TRLC

A diverse set of 5 machine learning algorithms and linear regression with 4 different types of regularization are evaluated and compared in their performance to predict *k*. The algorithms chosen were selected based on outstanding learning performance in retention time prediction as well as in general in the machine learning community and for their ability to perform feature selection [34]. The models are linear regression (LR) and SVR with a linear kernel, which both combine features to obtain the final prediction by assuming a linear relationship between the input variables and the output variable, and tree-based ensemble algorithms (RF, GB, XGBoost and OXT), which can capture complex non-linear relationships in the data by hierarchically combining features. Each model is firstly fine-tuned through hyperparameters search with Bayesian parameter optimization [35]. After optimization, 5-fold cross-validation (CV) of each model with the optimal parameters is performed and the results are used to compare the models. The metrics used are Pearson correlation coefficient (*r*) and mean absolute error (MAE). Pearson correlation coefficient measures the linear correlation between predicted and real values, it is calculated with the formula in equation 2. The MAE is calculated as in eq. 3.

2 $$\begin{aligned} r= & {} \frac{ \sum \limits _{i=1}^{n}(y _{real}-\bar{y}_{real})(y_{pred}-\bar{y}_{pred}) }{\sqrt{\sum \limits _{i=1}^{n}(y _{real}-\bar{y} _{real})^2}\sqrt{\sum \limits _{i=1}^{n}(y_{pred}-\bar{y}_{pred}})^2} \end{aligned}$$

3 $$\begin{aligned} MAE= & {} \frac{\sum \limits _{i=1}^{n}|y_{pred} - y _{real}|}{n} \end{aligned}$$

where $\hbox {y}_{{pred}}$ and $\hbox {y}_{{real}}$ are respectively the predicted and experimental values and *n* is the number of data points. The train and test split in 5-fold CV are 80:20. Out of the 1654 MDs, the ones producing noise (MDs that amount for low importance) are removed and the model is refitted only with the most important MDs [36], which corresponds to the ones explained in the results. Non-parametric statistical tests were implemented to compare the models because they are more robust and flexible than parametric tests. In this case, the Friedman test was first applied, followed by the post-hoc Nemenyi test. The Friedman test compares the medians of three or more groups. If the *p*-value is not significant, the medians of the groups are equal [37], otherwise, the median of at least one group is different. The next step is to use the Nemenyi post-hoc test to determine the pairwise group differences in the groups. The code to generate the models, and produce the figures was conducted in Python version 3.8. The overall workflow is represented in Fig. 2.

## Results and discussion

### Feature selection

**Fig. 3:**
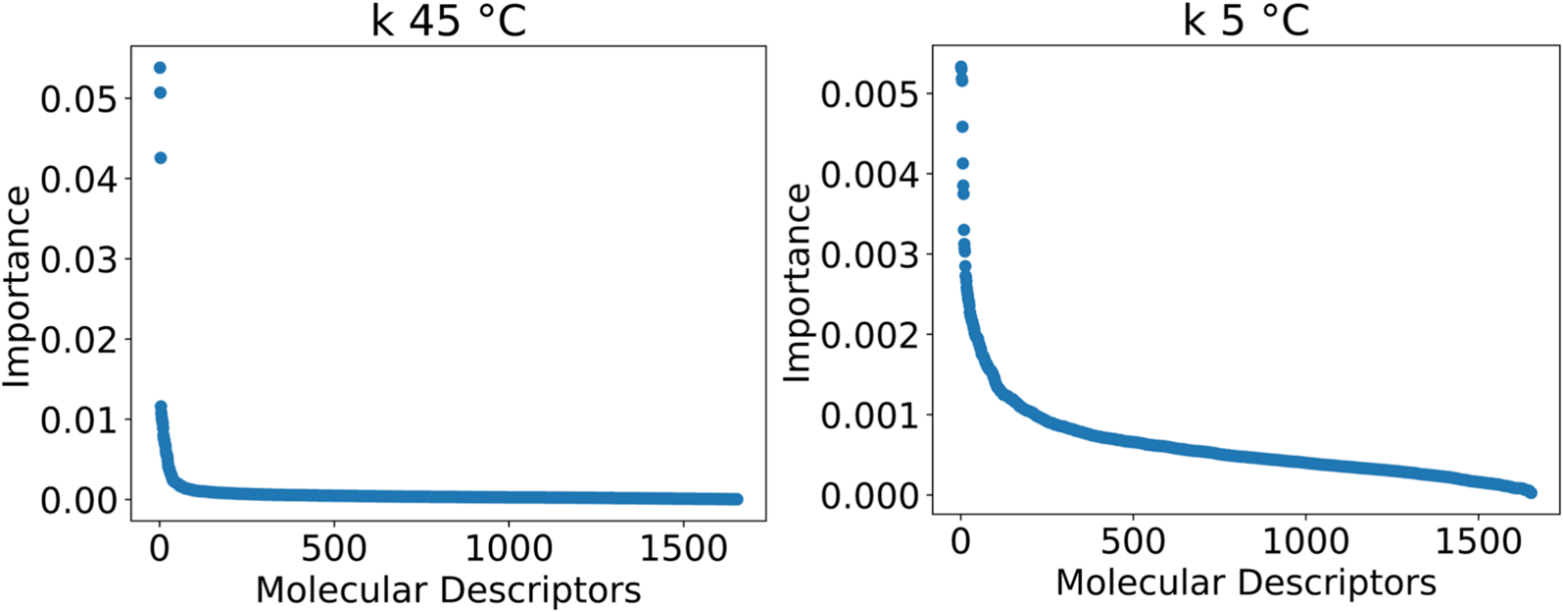
MDs importance normalized and averaged between all the models

**Fig. 4:**
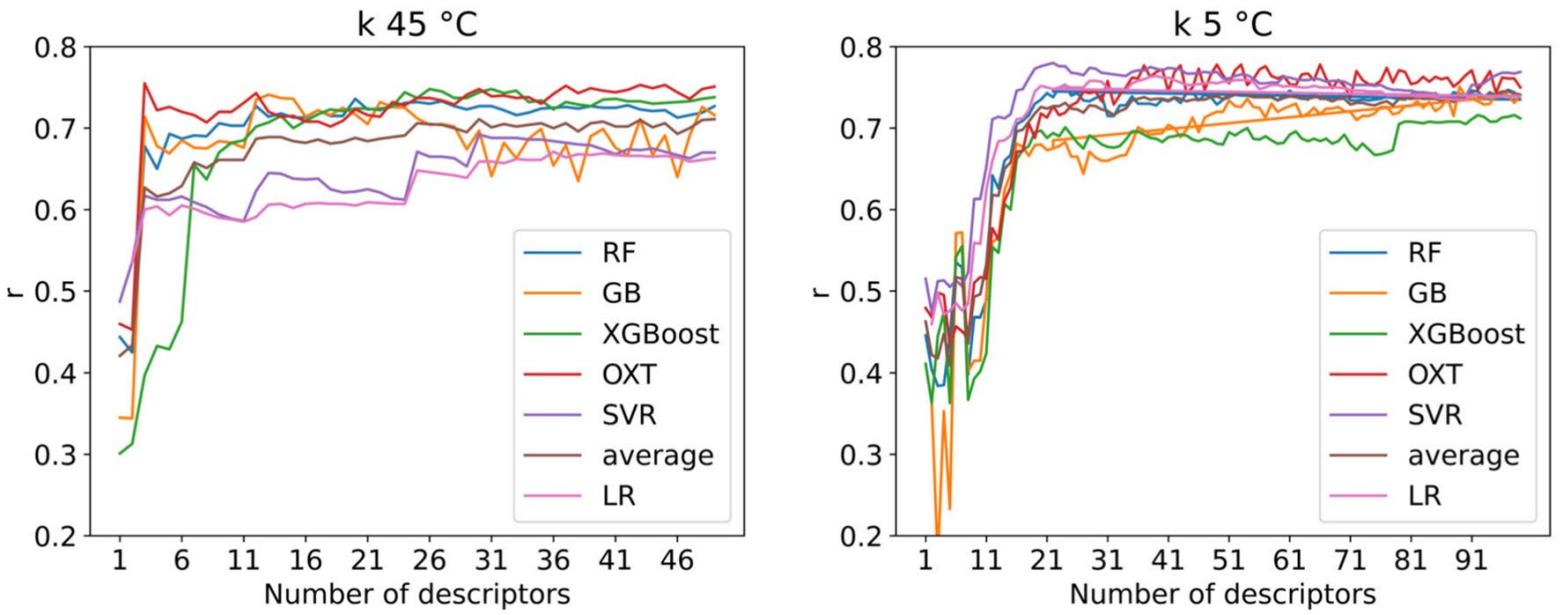
*r* of the test set depending on the number of MDs used to train the models

The MDs dataset is pre-processed as described in the experimental section, and the remaining 1654 MDs are fed to each model equally. The hyperparameters of the models are optimized on such dataset. All the models optimized are then tested on the same data over a 5-fold CV and evaluated with the same metrics. While linear regression and SVR with linear kernel give weights to the models’ features, tree-based ensemble models give importance based on metrics such as the mean decrease in impurity, which is the average reduction of the splitting criterion (such as Gini index or entropy) across all the trees in the ensemble [38]. In general, the ranking of feature importance may change as the number of MDs increases, especially if new features capture additional relevant information or interactions. For this reason, the contribution of each MD to the models is first scaled between 0 and 1 for each model and then averaged across all the models and scaled again. In this way, it is possible to have a total contribution of each MD to the retention. The high quantity of molecular descriptors used to train the models caused many features to be irrelevant and these are considered noise to the data. Figure 3 shows the plot of the MD’s importance, where the line flattens when the features become negligible and have little or no impact on the models. It was empirically estimated from the graphs that 62% of the MDs’ importance in the case of *k* at $45\,^{\circ }$C and 78% for *k* at $5\,^{\circ }$C were noise. Even though the noise portion accounts for a relatively high percentage of the total importance, the importance of each single descriptor is very low and, hence, not significant. The total number of relevant MDs results in 50 for *k* at $45\,^{\circ }$C and 100 in the case of *k* at $5\,^{\circ }$C. In the case of *k* at $45\,^{\circ }$C, the first three MDs’ importance accounted for 39% of the importance of the 50 MDs selected. Meaning that almost half of the model is based on 3 descriptors only. On the other hand, the non-noise MDs for *k* at $5\,^{\circ }$C had similar and smaller importance values, indicating a more complex and balanced mechanism that depends equally on many physicochemical parameters. To evaluate precisely the ideal number of MDs to use, the models are run over an increasing number of descriptors from 1 to either 50 or 100 depending on the *k* to predict. Figure 4 reports the change in *r* on the test depending on the number of MDs. The same plots also for *r* on the training set and MAE are reported in the supporting information section S3. Linear regression results are only presented for the regularization with the best overall performance (Ridge). For *k* at $45\,^{\circ }$C, there is a clear jump in performance when using 3 descriptors, where the highest point is reached with *r* of 0.75 for OXT. After that point, the performance oscillates around that value when increasing the number of MDs. In the case of *k* at $5\,^{\circ }$C, the increase in performance is less sharp and more gradual until 22 descriptors where the *r* is 0.78 for SVR and it remains almost constant after this point.

### Models performance and comparison

**Fig. 5:**
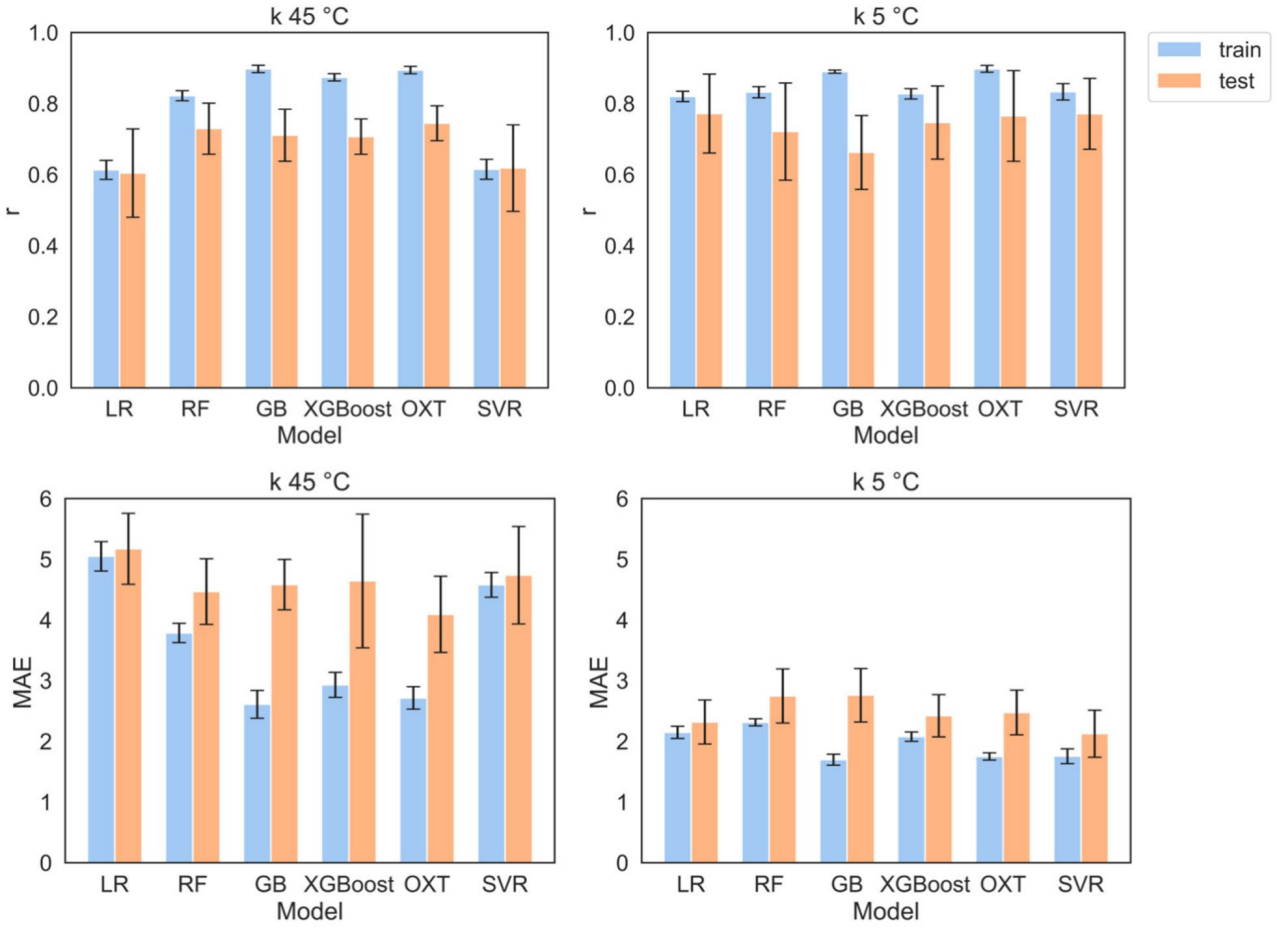
Comparison of the metrics used to evaluate the models for all the models tested across 5-fold CV

All the models tuned showed similar performance for both temperature cases ($45\,^{\circ }$C and $5\,^{\circ }$C) when trained with at least the minimum significant number of MDs, that is 3 for *k* at $45\,^{\circ }$C and 22 for *k* at $5\,^{\circ }$C. The CV averaged values of *r* on the test set vary between 0.6 and 0.74 for *k* at $45^{\circ }$C and 0.66 to 0.77 for *k* at $5\,^{\circ }$C, indicating good estimation (the goodness of fit plots and accuracy between predicting and actual retention time can be found in supporting information S4). Multiple linear regression without regularization failed to capture the complexity of the relationship between *k* and the MDs. However, regularization helped to prevent overfitting by adding a penalty term to the loss function. The penalty term reduces the magnitude of the coefficients making the model simpler and less prone to capture noise in the data and improves the results to match those of the other models. The prediction models for $5\,^{\circ }$C had slightly better performance than those for $45\,^{\circ }$C, as evidenced by the higher *r* values on the test, suggesting more precise predictions at $5\,^{\circ }$C. This can come from the narrower peak shapes at low temperatures as the compounds are generally less retained. At $45\,^{\circ }$C, more compounds are highly retained, consequently, they show increased peak width and lead to a higher error in the calculation of *k*. Other reasons for this difference in performance can arise from the fact the dataset for $45\,^{\circ }$C is more spread, again due to the higher retention (supporting information Fig. 8), or because of external factors that are not taken into account in the models such as the dielectric constant of the water, that assumes different values depending on the temperature. Figure 5 summarizes the evaluation of the models in terms of *r* on both train and test and MAE on the average of 5-fold CV for *k* at 5 and $45\,^{\circ }$C (tables containing the data are available in supporting information S5). Consideration of the MAE requires attention to the range of *k* values observed. Specifically, at $45^{\circ }$C, the highest *k* value stands at 53.6, attributed to bisphenol A, whereas at $5^{\circ }$C, naproxen demonstrates the highest *k* of 32.8. To capture differences depending on the model, the Friedman and post-hoc Nemenyi tests were performed on the test set. The results obtained showed that for *k* at $45\,^{\circ }$C there is no significant difference between the models in terms of *r*. When looking at MAE similar results are achieved, except for OXT, which outperforms LR. In the case of *k* at $5\,^{\circ }$C the results are also that the performances of the models are very similar, except SVR which exceeds RF and GB in terms of MAE. Nonetheless, the *p*-values were relatively close to the significantly different threshold (see SI section S6), suggesting that overall, the performance of all the models across metrics and temperatures is very similar. For this reason, the importance of the MDs is averaged across all models. Finally, the applicability domain (AD) was also determined to assess the absence of outliers in the dataset and the chemical space for which the models are valid. The AD was determined using the leverage approach [39], and the relative Williams plot can be found in SI section S7.

### Physicochemical elucidation of TRLC retention mechanisms at high temperatures

**Fig. 6:**
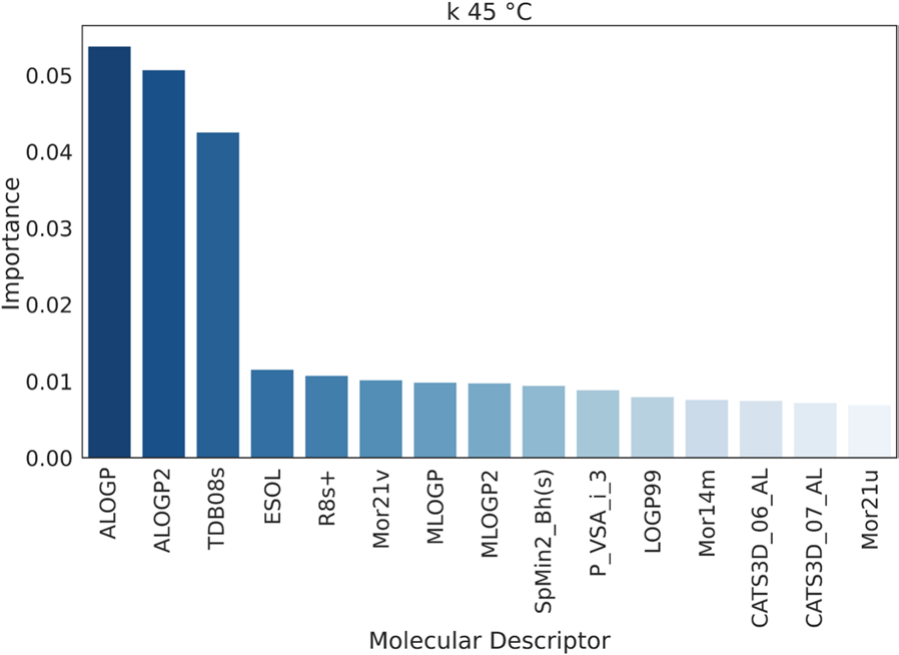
15 most influential MDs for *k* at $45\,^{\circ }$C mechanism and their relative importance

At $45\,^{\circ }$C, the polymer is in the collapsed form, the monomer’s side chains form bonds between each other and, consequently, the amine is shielded by the polymer backbone and the isopropyl group is exposed. In this situation, we observe the RPLC-type of the retention mechanism. A plot of the first 15 more important MDs with relative importance is represented in Fig. 6, all the most important MDs and their respective explanations can be found in SI section S8 for both temperatures. The physicochemical parameters that dominate the models at high temperatures are indeed mostly related to logP, which is the main parameter determining retention in RPLC. LogP is an indicator of hydrophobicity, which is a measure of the difference in free energy of a molecule in the two phases, octanol, and water. This difference depends on three factors: the enthalpy of interaction between the solute and the solvent, the enthalpy of interaction between solvent molecules, and the entropy changes that arise from the change in solvent structure around the solute. Each atom in a molecule interacts differently with the surrounding solvent based on its electronic distribution and approachability. More hydrophilic molecules have a higher affinity to the aqueous mobile phase and elute faster than hydrophobic molecules [40]. For instance, the introduction of carbon substitution enhances hydrophobicity, subsequently boosting retention. Conversely, the inclusion of a single heteroatom decreases both hydrophobicity and retention. However, this pattern reverses when multiple heteroatoms are present. This can be seen in benzene and toluene: toluene has one carbon in the benzene ring substituted by a -CH3 group and, as predicted, the retention is increased (k $45\,^{\circ }$C benzene = 1.7, *k* $45\,^{\circ }$C toluene = 2.9). One group worth mentioning are the sulfur-containing compounds. Divalent sulfurs are by nature hydrophobic, while sulfuryl and sulfonyl sulfurs are weakly hydrophilic. Indeed, the sulfonamide antibiotics in the dataset, that have a sulfonamide group (R-SO2-NH2, divalent sulfur) attached to a benzene ring are all retained at $45\,^{\circ }$C. Some other compounds containing hexavalent sulfur were also analyzed and showed retention despite being weakly hydrophilic, however, this can be explainable by the presence of other heteroatoms in the molecules. LogP is present in forms of different calculations such as ALOGP, MLOGP, their squared forms and LOGP99. The 3 most important MDs that allow to reach good performance with most of the models include ALOGP, ALOGP2 and TDB08s. The latter is a topological autocorrelation descriptor calculated for the distance range 8 and weighted by the I-state, which is a quantification of the electronic and topological environment of the atom considered. A high value means that the molecule has many bonds of different lengths and types. TDB08s is not the only descriptor related to the I-state, indeed there is also R8s+, which corresponds also to the autocorrelation in the same lag 8, and SpMin2_Bh(s). Solubility (ESOL) is also amongst the more relevant descriptors, having a similar meaning to logP. In this same fashion, there are some MDs that describe the hydrogen bond acceptor interactions (CATS3D), specifically the presence of acceptor-lipophilic points at specific distances has an impact on retention. The presence of these descriptors in the lower range of importance can highlight the possibility of interactions with residual silanol and amino groups on the surface of the stationary phase. Even though the column is end-capped, there is still a chance that not all the aminopropyl groups react. Therefore, the retention is increased by a higher number of hydrogen-acceptor atoms that are retained as a consequence of the interaction with the groups still present in the silica. The presence of MDs that account for the ionization potential (p_VSA_i_3) of the molecule hints at possible dipole interactions also happening. Under the analysis conditions used, the mobile phase constituted of water with 0.1$\%$ FA, the pH inside the column is close to 2, and at this pH value, the stationary phase is protonated. A positive layer could form that causes the increase in retention for molecules with high values of negative charge. The presence of descriptors that account for the volume occupied by the molecule and the mass show that also the size is relevant. The least retained compound in the dataset used is thymine, with a *k* of 0.14, and it indeed shows a low value of logP (ALOGP = -0.6), no acceptor group, and a compact structure. While the most retained compound, bisphenol A, with *k* = 53.6, has a higher value of logP (ALOGP = 3.7), multiple acceptor groups, and high ionizability on the surface area, all parameters that suggest high retention. The following list summarizes the interactions that govern retention in TRLC at high temperatures: (i) hydrophobic interactions with the isopropyl groups of the polymer, (ii) hydrogen acceptor/donor interactions with the silanol and aminopropyl unreacted groups from the silica, iii) dipole interactions between the silica positively charged layer and molecules with high values of electronegativity.

### Physicochemical elucidation of TRLC retention mechanisms at low temperatures

**Fig. 7:**
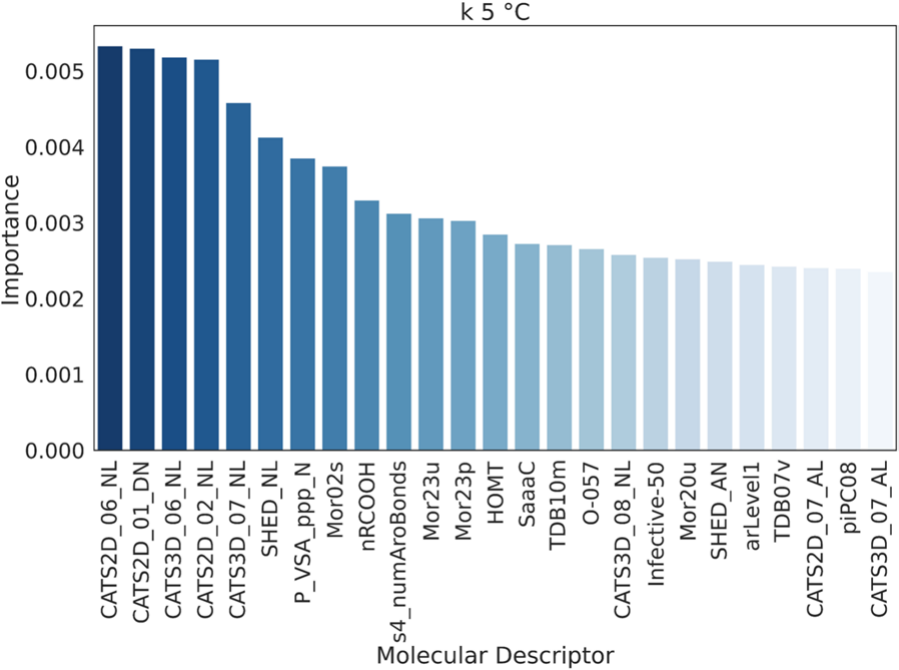
25 most influential MDs for *k* at $5\,^{\circ }$C mechanism and their relative importance

At $5\,^{\circ }$C, below the polymer LCST, the side chains are swelled in the water, and they bond with the $\hbox {H}_2$O molecules of the mobile phase. The mechanism at low temperatures seems much more complex than at high temperatures, as no dominant feature can explain it (Fig. 7). It does not resemble any more RPLC, and it is almost equally dominated by many physicochemical parameters, leading to the thought that multiple and complex interactions happen. The silica is less accessible by the analytes and the polymer side chains are protonated and available to form bonds with the compounds passing through the column. At low temperatures there are still hydrogen-acceptor-related descriptors, however, there are also hydrogen-donor MDs. The presence of both donor and acceptor groups in the polymer can lead to thinking that both interactions happen, with the =O as an acceptor and -NH as a donor. Certainly, the presence and distribution of negative lipophilic points are also very influential. Potential negative groups are all the groups in a molecule that can have a partial or full negative charge, such as oxygen in hydroxyl or carboxyl groups, or halogens. Generally, molecules with too many or too few negative charges are less retained, while molecules with a moderate number of negative charges are retained more. These groups are described by SHED and CATS descriptors, which measure the density of the pharmacophoric points at different distances in the molecular structure. A high value of SHED indicates a complex and flexible molecule that can interact with the stationary phase more effectively. In our dataset, we observed that all the compounds with *k* at $5\,^{\circ }$C greater than 15, have a SHED_NL value greater than 4. Features that describe the complexity of the molecule in terms of structure and functional groups appear to be more relevant at low temperatures than at high temperatures. Molecules that possess the structure of carboxylic groups, aromatic rings, and $\hbox {sp}_2$ hybridized carbons show higher retention, while the presence of oxygen groups such as phenol or enol decreases the retention. This can be observed, for example, in the compound folinic acid, which has a high retention at $5\,^{\circ }$C (k = 18.0) due to the presence of an aromatic ring in the structure and two carboxylic acid groups. The more balanced situation of quercetin, with two benzene rings but also many phenolic oxygens, explains its moderate retention (k = 7.8). Furthermore, mass and volume-related MDs are relevant for *k* at $5\,^{\circ }$C in the same fashion as for *k* at $45\,^{\circ }$C, hence, at low temperatures as well, the molecule size matters. Interestingly, at low temperatures, MDs that describe chirality are present, which are also related to aromatic bonds. Therefore, the presence of aromatic structures seems to increase the retention especially if they are close to a chiral centre. The compound that shows higher retention at $5\,^{\circ }$C in our dataset is naproxen with a *k* of 32.8. It matches the mechanism description, having most of the features that correlate to an increase in retention such as the high number of aromatic bonds at a chiral centre substituent, that are also benzene rings, the presence of a -COOH, and a high value of SHED_NL, just to mention few. This is also confirmed by the least retained compound, thymine, *k* = 0.2, where there are no chiral centres, no aromatic rings, no carboxylic acids, null value of SHED_NL, and only a few carbons hybridized $\hbox {sp}_2$ that could guarantee the compound the little retention. For most of the molecules, we expect a decrease in retention with increased temperature, however, this is not always the case. Many molecules in the dataset show no difference in *k* with temperature and some are even more retained at low temperature. This is clearer to explain as the two mechanisms are elucidated. For example, antipyrine is barely retained at $45\,^{\circ }$C (k = 1.8), indeed the logP is relatively low (ALOGP = 1.6), it presents only one possible acceptor atom in the structure, while at $5\,^{\circ }$C is more retained (k = 6.3), in line with the presence of the benzene ring, and multiple $\hbox {sp}_2$ hybridized carbons. To summarize the retention mechanism at $5\,^{\circ }$C: i) donor/acceptor interactions with the groups in the polymer side chains, ii) weak interactions with the silica, iii) structure and functional group-related interactions.

## Conclusions

In this work, the retention mechanisms of temperature-responsive liquid chromatography are studied through the feature selection of the most common machine learning algorithms for prediction models. After evaluating the predictability of an in-lab-created dataset of 139 molecules analysed with TRLC at high and low temperatures, the molecular descriptors used by the machine learning algorithms were evaluated and explained with relevance to the retention mechanism. Different models were tested, including linear regression, SVR, and tree-based ensemble models, and there was no outstanding one in terms of performance. The metrics used, together with the statistical analysis, validate the precision, accuracy, and robustness of the models. Each algorithm used has a selection method implemented that allows the establishment of which features impact the predictability of the variable. The evaluation of the models’ most important MDs made it possible to understand what physicochemical parameters drive the retention mechanism at 45 and $5\,^{\circ }$C. At $45\,^{\circ }$C, the column seems to behave similarly to reversed-phase columns, where the retention is primarily dictated by logP. At $5^{\circ }$C it looks much more complex, there is no unique influence of an MD, however, there is a recurrent characteristic of the more important MDs: they are mostly about the negative and lipophilic nature of the molecular structure and many also related to the presence of specific functional groups. While it is not possible to conclude that there is a trend in retention directly related to the presence/absence of such points in a compound, it is observable that some specific combination (e.g., a high number of negative points, and the presence of many sparse negative-lipophilic points on the molecule surface) are associated with an increase or a decrease in retention. The elucidation of the mechanism led to the hypothesis that there are interactions with the unreacted aminopropyl and silanol groups in the silica, a better control during manufacturing of the elimination of these groups could open the possibility of obtaining better chromatography in terms of selectivity and peak shape. While this work focus was on the most used temperature-responsive polymer, there are others such as PDEAAm (Poly(N, N-dimethyl acrylamide)), which have a very similar structure to PNIPAAm with the difference that the nitrogen on the side chain has three substituents, hence no available hydrogen. A future modelling study on this polymer could give more insight into the retention mechanism of TRLC, and also prove the interactions that are due to the -NH of PNIPAAm. In this framework the effect of the solvent was not considered, however, looking into the dielectric constant of water at different temperatures and surface tension can also improve the prediction and give insights into the possible presence of ionic interaction.

### Supplementary Information

Supplementary Material 1.

## Data Availability

The dataset supporting the conclusions of this article is included in the article’s SI. The code developed for this work is freely available at https://github.com/ebandini/TRLC_prediction.

## Abbreviations

TRLC: Temperature-responsive liquid chromatography
HPLC: High-performance liquid chromatography
LCST: Lower critical solution temperature
PNIPAAm: Poly-N-isopropyl acrylamide)
RPLC: Reversed-phase liquid chromatography
MD: Molecular descriptor
RF: Random Forest
OXT: Extra Trees Regressor
GB: Gradient Boosting
XGBoost: Extreme Gradient Boosting
SVR: Support Vector Regression
ANNs: Artificial Neural Networks
FA: Formic acid
MAE: Mean absolute error
CV: Cross validation
AD: Applicability domain
PDEAAm: Poly(N, N-dimethyl acrylamide
LR: Linear regression

## Code availability

The code developed for this work is freely available at https://github.com/ebandini/TRLC_prediction.
