# Supplementary material for: Physicochemical modelling of the retention mechanism of temperature-responsive polymeric columns for HPLC through machine learning algorithms
Source: J Cheminform. 2024 Jun 21;16:72. doi: 10.1186/s13321-024-00873-6 (PMC11193285; doi:10.1186/s13321-024-00873-6)
Supplement: Supplementary file 1 — Supplementary Material 1. [file 13321_2024_873_MOESM1_ESM.pdf]

S1. List of molecules

All the solutes used in this work (full list in Table 3) are from Sigma-Aldrich (Steinheim, Germany), UCB (Brussels, Belgium), Fluka (Buchs, Switzerland), Texaco (Ghent, Belgium), Acros (Geel, Belgium), Janssen Chimica (Beerse, Belgium). Retention factor k was calculated as follows (Equation 4):

$$k = \frac{t_r - t_0}{t_0} \tag{4}$$

where t_R is the retention time and t_0 is the column dead time. Figure 8 shows the distribution of the data for both k temperatures.

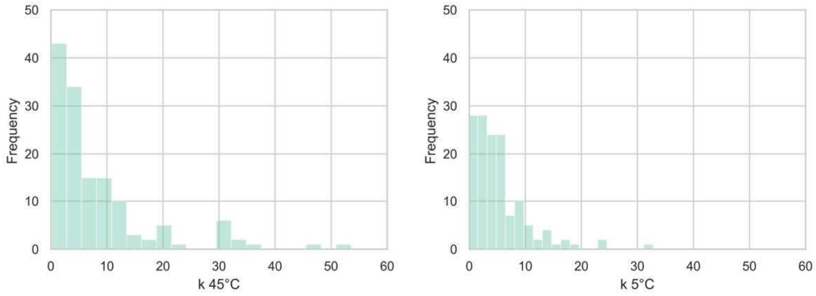

Fig. 8 Histograms of the data distribution for k at 45 °C and k at 5 °C.

Table 2: Full list of compounds used to train and test the models with respective k at 45 and 5 °C, ordered by descending value of k at 45 °C. The compounds with an * are also confirmed by HRMS analysis.

Compound name	CAS	k at 45°C	k at 5°C	UV wavelength (nm)
bisphenol A	80-05-7	53.55	14.26	254
mecoprop-P	16484-77-8	45.85	0.00	254
4-tert-butylbenzoic acid	98-73-7	35.44	17.53	254
estrone	53-16-7	33.36	13.18	254
cefamandole*	42540-40-9	33.05	23.8	192
equiline	474-86-2	31.67	14.52	254
ibuprofen	15687-27-1	31.63	11.18	254
naproxen	22204-53-1	30.41	32.77	254
mesterolone	1424-00-6	30.30	6.24	254
ketoprofen	22071-15-4	30.03	23.87	254
progesterone	57-83-0	29.96	12.88	254
purpurin	81-54-9	22.72	0.00	254
MCPB	94-81-5	21.32	15.27	254
butylparaben	94-26-8	19.57	8.15	254
17-α-hydroxyprogesterone acetate*	302-23-8	19.42	5.52	254
sulfaquinoxaline	59-40-5	19.30	16.80	254
dehydro epiandrosterone	53-43-0	19.30	6.23	254
5,5-diphenylhydantoin	57-41-0	16.93	8.69	258
norethisterone	68-22-4	16.71	6.10	240
testosterone	58-22-0	15.96	5.67	254
hexanophenone	942-92-7	14.34	8.99	254

Continued on next page

Table 2: Full list of compounds used to train and test the models with respective k at 45 and 5 °C, ordered by descending value of k at 45 °C. The compounds with an * are also confirmed by HRMS analysis. (Continued)

Compound name	CAS	k at 45°C	k at 5°C	UV wavelength (nm)
BHA (3-tert-Butyl-4-hydroxyanisole)	121-00-6	13.58	5.37	254
2,7-dihydroxynaphthalene	582-17-2	12.89	12.25	254
myclobutanil	88671-89-0	12.59	6.58	254
Androsta-1,4-dien-3-one-17β-hydroxy-17-methyl	72-63-9	12.55	4.35	254
estriol	50-27-1	12.51	5.10	215
ethisterone	434-03-7	12.35	4.79	240
propylparaben	94-13-3	11.88	5.77	254
2,4-dichlorophenol	120-83-2	11.85	9.20	254
exemestane	107868-30-4	11.54	5.86	254
indole-3-butyric acid	133-32-4	11.44	10.27	254
folinic acid*	1492-18-8	11.04	17.98	254
naphthalene	91-20-3	10.81	13.21	254
linuron	330-55-2	10.72	10.25	254
nandrolone	434-22-0	10.38	4.14	254
propyl gallate	121-79-9	10.37	7.69	215
tert-butylhydroquinone	1948-33-0	10.07	4.33	280
rutin	153-18-4	9.77	9.02	254
diphenyl carbonate	102-09-0	9.75	6.07	254
(+)-catechin	7295-85-4	9.68	9.39	254
terbuthylazine	5915-41-3	9.56	5.51	254
(+)-griseofulvin	126-07-8	9.38	9.96	254

Continued on next page

Table 2: Full list of compounds used to train and test the models with respective k at 45 and 5 °C, ordered by descending value of k at 45 °C. The compounds with an * are also confirmed by HRMS analysis. (Continued)

Compound name	CAS	k at 45°C	k at 5°C	UV wavelength (nm)
α-resorcylic acid	99-10-5	9.13	7.97	254
4-tert-butylphenol	3101-60-8	9.07	3.73	254
rifampicin*	13292-46-1	9.00	6.45	254
butyl phenyl ether	1126-79-0	8.95	6.09	254
quercetin	117-39-5	8.72	7.81	254
valerophenone	1009-14-9	7.85	5.97	254
naringin	480-41-1	7.81	8.55	254
1,2,4-trimethyl benzene	95-63-6	7.61	5.66	240
cumene	98-82-8	7.37	5.81	254
betamethasone	378-44-9	7.08	2.98	254
toluic acid	99-94-5	6.73	6.36	254
isoeugenol	97-54-1	6.38	5.59	270
aspirin	50-78-2	6.35	9.19	254
sulfamethoxazole	723-46-6	6.35	5.82	254
sebuthylazine	7286-69-3	6.31	3.81	254
(-)-epicatechin	490-46-0	6.29	9.75	254
benzoin methyl ether	3524-62-7	5.97	5.08	254
ethyl paraben	120-47-8	5.69	3.73	254
diphenylmethanol	91-01-0	5.59	3.97	254
hippuric acid	495-69-2	5.58	8.84	254
5,7-dimethoxycoumarin*	487-06-9	5.45	5.76	254
carbamazepine	298-46-4	5.14	8.44	254
alachlor	15972-60-8	5.14	3.25	254
4-hydroxy benzoic acid	99-96-7	4.96	3.54	254

Continued on next page

Table 2: Full list of compounds used to train and test the models with respective k at 45 and 5 °C, ordered by descending value of k at 45 °C. The compounds with an * are also confirmed by HRMS analysis. (Continued)

Compound name	CAS	k at 45°C	k at 5°C	UV wavelength (nm)
2-ethoxybenzoic acid	134-11-2	4.95	7.97	254
cyanazine	21725-46-2	4.94	3.28	254
syringic acid	530-57-4	4.89	5.32	254
3,4-dihydroxy benzoic acid	99-50-3	4.88	4.17	325
isoproturon	34123-59-6	4.73	5.39	254
vanillic acid	121-34-6	4.72	4.08	254
corticosterone	50-22-6	4.68	2.53	254
piroxicam	36322-90-4	4.67	7.90	254
butyrophenone	495-40-9	4.64	4.14	254
hydrochlorothiazide	58-93-5	4.51	4.92	275
3-(2-furyl) acrylic acid	539-47-9	4.47	4.62	254
sulfamethizole	144-82-1	4.35	4.19	254
eugenol	97-53-0	4.01	3.86	270
diethyl phthalate	84-66-2	3.92	3.60	228
3,4-dichloro aniline	95-76-1	3.65	0.96	254
1-chloro-4-nitrobenzene	100-00-5	3.53	4.53	254
4-(dimethylamino)benzoic acid	619-84-1	3.47	3.78	254
o-methyl anisole	578-58-5	3.37	3.28	210
methylparaben	99-76-3	3.36	2.56	254
3-nitrophenol	554-84-7	3.36	2.33	266
prednisone	53-03-2	3.19	1.67	254
cortisone	53-06-5	3.19	1.61	254

Continued on next page

Table 2: Full list of compounds used to train and test the models with respective k at 45 and 5 °C, ordered by descending value of k at 45 °C. The compounds with an * are also confirmed by HRMS analysis. (Continued)

Compound name	CAS	k at 45°C	k at 5°C	UV wavelength (nm)
pyridine	110-86-1	3.09	0.00	254
trans-ferulic acid	537-98-4	2.95	1.87	254
propiophenone	93-55-0	2.94	2.98	254
tropolone	533-75-5	2.91	2.67	280
toluene	108-88-3	2.89	2.72	227
(+)-carvone	6485-40-1	2.85	3.95	262
cinnamaldehyde	104-55-2	2.85	4.63	285
abiraterone*	154229-19-3	2.83	0.34	255
L-(-)-carvone	6485-40-1	2.80	3.69	315
sulfathiazole	72-14-0	2.73	2.99	254
methyl benzoate	93-58-3	2.67	2.99	254
m-cresol	108-39-4	2.65	2.09	254
3-(5-methylfuran-2-yl)-propionic acid	1456-08-2	2.64	2.12	254
nortriptyline	894-71-3	2.46	-0.13	254
cinnamyl alcohol	104-54-1	2.28	2.45	254
trans-2-hexenyl acetate	2497-18-9	2.25	0.97	254
camphor*	76-22-2	2.08	1.72	280
anisole	100-66-3	2.07	2.62	210
vanillin	121-33-5	1.98	2.26	280
tropic acid	552-63-6	1.97	2.12	210
nitrocyclohexane	1122-60-7	1.91	2.19	254
dimethyl phthalate	131-11-3	1.89	2.15	224
antipyrine*	60-80-0	1.85	6.30	205
acetophenone	98-86-2	1.79	1.93	254

Continued on next page

Table 2: Full list of compounds used to train and test the models with respective k at 45 and 5 °C, ordered by descending value of k at 45 °C. The compounds with an * are also confirmed by HRMS analysis. (Continued)

Compound name	CAS	k at 45°C	k at 5°C	UV wavelength (nm)
benzene	71-43-2	1.69	1.63	254
gibberellic acid	77-06-5	1.66	0.51	254
phenol	108-95-2	1.62	1.55	254
1,3-butanediol diacrylate	19485-03-1	1.54	1.01	254
sulfadiazine	68-35-9	1.53	1.28	254
atrazin-desethyl-2-hydroxy	645-92-1	1.52	1.40	254
phthalide	1135443-46-7	1.46	2.31	254
sulfamerazine	127-79-7	1.45	0.99	254
4-Phenyl-1,3-Dioxane	99494-18-5	1.41	1.43	254
p-benzoquinone	106-51-4	1.36	1.08	254
sulfacetamide	144-80-9	1.33	1.12	254
catechol	120-80-9	1.25	1.17	254
aldicarb*	116-06-3	1.01	1.28	254
acetaminophen	103-90-2	0.98	1.08	244
pyrogallol	87-66-1	0.97	1	254
benzamide	55-21-0	0.95	1.48	254
pentoxifylline	6493-05-6	0.93	2.73	275
formononetin	485-72-3	0.89	2.37	254
thiamethoxam	153719-23-4	0.83	2.26	254
benzyl alcohol	100-51-6	0.82	0.64	254
metamitron	41394-05-2	0.78	1.13	254
riboflavin	83-88-5	0.51	1.01	245
sulfaguanidine	57-67-0	0.44	0.45	254

Continued on next page

Table 2: Full list of compounds used to train and test the models with respective k at 45 and 5 °C, ordered by descending value of k at 45 °C. The compounds with an * are also confirmed by HRMS analysis. (Continued)

Compound name	CAS	k at 45°C	k at 5°C	UV wavelength (nm)
L-(+)-ascorbic acid	50-81-7	0.38	0.29	254
caffeine	58-08-2	0.35	0.54	254
2-hydroxy pyridine	142-08-5	0.34	0.82	295
thymine	65-71-4	0.14	0.23	254

S2. Temperature-responsive polymer characterization

The PNIPAAm polymer was synthesized following the procedure explained by Baert et al. [9]. The polymerization was checked via GC on an Agilent 7890A GC-FID system equipped with an HP5-MS column (30 m x 0.25 mm x 0.25 μm) (Agilent). The inlet and outlet temperatures were set at 300 °C. Hydrogen, supplied by a VWR Carrier-160 hydrogen generator, was used as a carrier gas and mobile phase velocity was 1.2 mL min^{-1}. Split-injection was used with a split ratio of 20:1. The oven temperature was increased by 5 °C min^{-}1 from 35 °C to 65 °C, followed by a ramp of 25 °C min^{-1} to 300 °C. The conversion was determined by monitoring the change in the monomer to polymerization solvent concentration. A conversion of 88% is reached and a yield of 81%. The molecular weight of the polymer was measured by Size Exclusion Chromatography (SEC) with light scattering detection. The results of the analysis are Mw = 12630 g mol^{-1} and polydispersity 1.552. SEC was done on an Agilent 1260-series HPLC system (Agilent, Waldbronn, Germany) with two PLgel 5 μmm columns (300 x 7.5 mm) and a mixed-D guard column (50 x 7.5 mm, Agilent) in series. The detector was a 1260 diode array detector, a refractive index detector and a multi-angle light scattering detector (MALS, WYATT technology, Santa Barbara, USA). The eluent used was dimethylacetamide with 50 mM lithium chloride at a

flow rate of 0.5 mL min^{-1}. The calibration is based on poly(methylmethacrylate) (PMMA) standards from Polymer Standard Service (Mainz, Germany). Cloud point temperature (TCP) is calculated from turbidimetry measurement on Crystal116T M parallel crystallizer developed by Avantium Technologies connected to a recirculation chiller and dry compressed air. The polymer was solubilized in water (5 mg mL^{-1}) and heated from 3 °C to 45 °C with a heating rate of 1 °C min^{-1} followed by cooling to 3 °C at the same rate. The cycle was repeated three times and the TCP is defined at 50% transmittance, TCP = 29.9 °C. A demonstration of the responsivity of TRLC columns is shown in Figure 9. The column used to produce the chromatograms is the same used for this work, the analyses are conducted at 45°C and 5°C for the compound in the dataset 4-tert-butylphenol. The analyte is retained at 45°C with $k = 9.1$, while the retention time decreases at 5°C, $k = 3.7$.

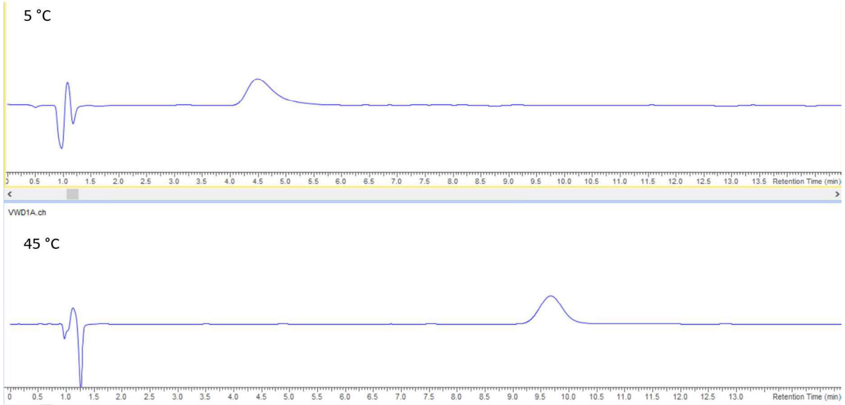

Fig. 9 Analysis of 4-tert-butylphenol at 5 °C and 45 °C with the temperature responsive column made of PNIPAAm polymer used in this work.

S3. Feature selection

The ideal number of MDs for the models is set as the minimum number of MDs for which we obtain the best values for the metrics used, r and MAE. The change in MAE

depending on the number of MDs is represented in Figure 10. For k at 45 °C, we can

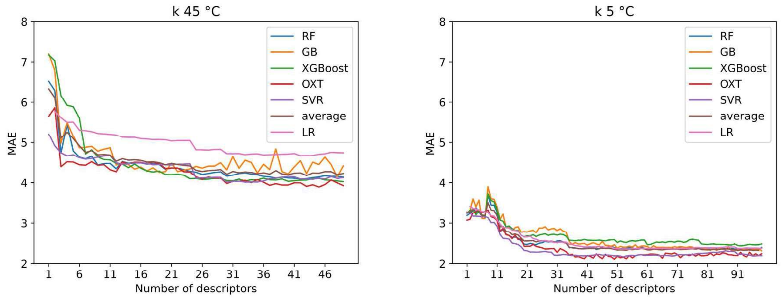

Fig. 10 r of the test set depending on the number of MDs used to train the models.

see the same jump, as with r, with MAE when using 3 descriptors, where the lowest point is reached with MAE of 4.1 for OXT. In the case of k at 5 °C, similarly as with r, also with MAE, the increase in performance, which corresponds to a decrease in MAE, is gradual and it starts to stabilize after 31 descriptors where the MAE is 2.3 for SVR, and it remains almost constant after this point.

S4. The goodness of fit

Prediction results for training and testing for each model are represented in figures 11 and 12 versus the real values. The same train/test split is considered for all the models in each temperature. It is noticeable in the case of k at 5 °C that the predictions for longer retention times mostly give a negative error, the predicted value is smaller than the experimental value. This can come from the negative skewed Gaussian shape given by the fronting that happens in the chromatographic peaks when they reside longer time in the column. This is less pronounced at 45 °C as the peaks tend to form also some extent of tailing, hence they increase the total peak width, consequently, the error in predictions is equal on both sides (positive and negative).

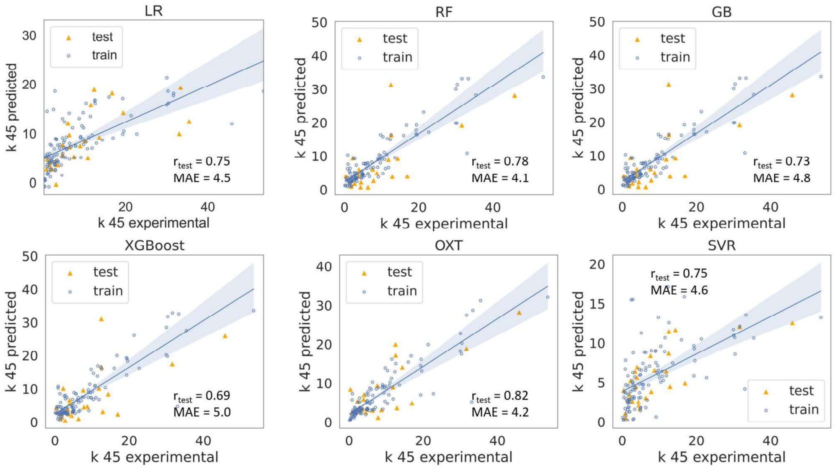

Fig. 11 Goodness of fit between predicted and real values for both train and test set of each model, predicting value k at 45 °C.

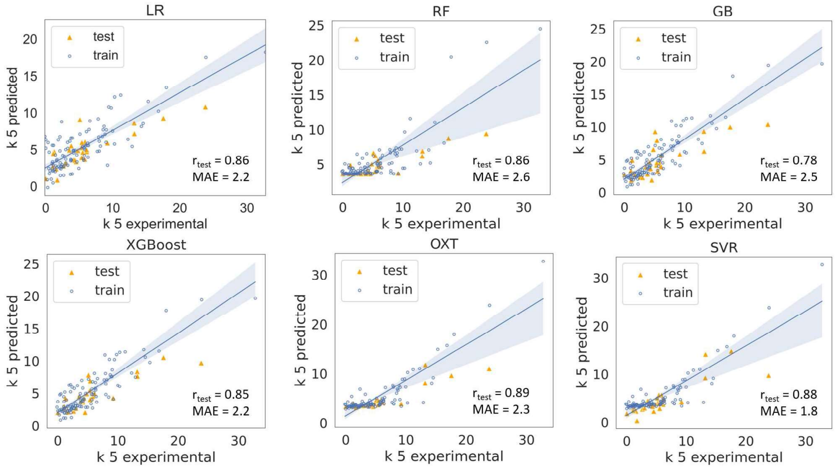

Fig. 12 Goodness of fit between predicted and real values for both train and test set of each model, predicting value k at 5 °C.

S5. Performance of the models

The performance of the models is summarized numerically in tables 3 and 4.

Table 3 Models' evaluation for prediction of k at 45 °C, results averaged over 5-fold CV.

Model	r on train set	r on test set	MAE
LR	0.60 ± 0.03	0.60 ± 0.13	5.6 ± 0.7
Random Forest	0.82 ± 0.01	0.72 ± 0.08	4.5 ± 0.5
Gradient Boosting	0.90 ± 0.01	0.71 ± 0.07	4.6 ± 0.4
XGBoost	0.87 ± 0.01	0.71 ± 0.05	4.6 ± 1.1
Extra Trees Regression	0.89 ± 0.01	0.75 ± 0.05	4.1 ± 0.6
SVR	0.61 ± 0.03	0.62 ± 0.12	4.7 ± 0.8

Table 4 Models' evaluation for prediction of k at 5 °C, results averaged over 5-fold CV.

Model	r on train set	r on test set	MAE
LR	0.77 ± 0.02	0.75 ± 0.15	2.7 ± 0.4
Random Forest	0.83 ± 0.01	0.73 ± 0.12	2.7 ± 0.4
Gradient Boosting	0.89 ± 0.01	0.66 ± 0.10	2.8 ± 0.5
XGBoost	0.83 ± 0.01	0.75 ± 0.10	2.4 ± 0.3
Extra Trees Regression	0.90 ± 0.01	0.77 ± 0.12	2.5 ± 0.4
SVR	0.80 ± 0.02	0.78 ± 0.13	2.3 ± 0.4

S6. Statistical tests

To find significant differences between models in the test set, the Friedman and post-hoc Nemenyi tests were applied to the r and MAE metrics for the 6 models. Results are shown in Figure 13.

S7. Applicability domain

The applicability domain defines the chemical space in which predictions are considered valid and consequently, it encloses the space where robust future predictions can be made. In this work, it was determined using the leverage approach. The leverages of each compound in the dataset are the diagonal elements of the hat matrix (H) calculated by the formula in equation 5, where X is the data matrix. The calculations are made using the models that delivered the best results, OXT for k at 45 °C and SVR for k at 5°C, and a restricted set of MDs, the first 3 most important for k at 45 °C and 22 for k at 5 °C.

$$H = X(X^T X)^{-1} X^T \tag{5}$$

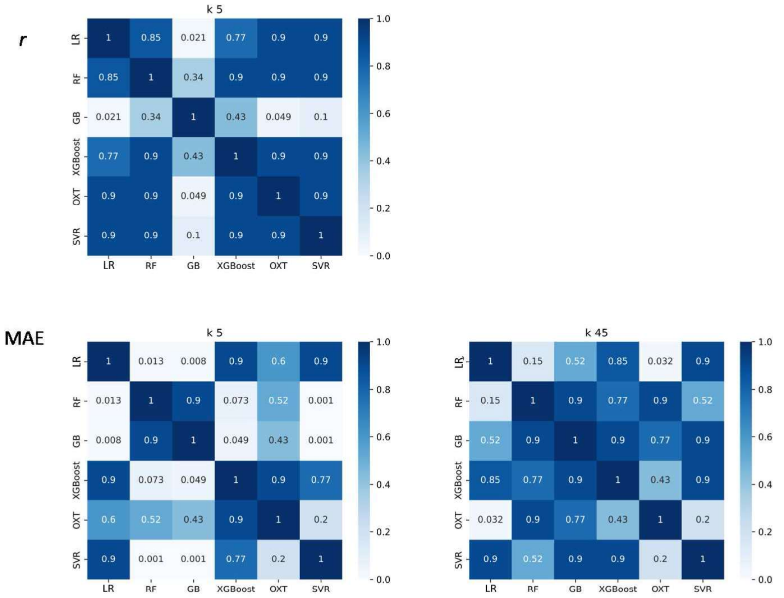

Fig. 13 Heat map of the p-values of the Nemenyi test for the metrics r and MAE. For k at 45 °C r is not shown as it delivers no significant difference in terms of the p-value from the Friedman test.

In figure 14, the standardized residuals are plotted versus the leverages (Williams plot). From this plot, the applicability domain is established inside the squared area defined by the threshold h* (equation 6)

$$h^* = \frac{2p}{n} \tag{6}$$

Where p is the number of features and n is the number of training compounds.

S8. Molecular descriptors

After excluding the noise, the remaining MDs for each temperature are reported in Tables 3 and 3 with their normalized importance and descriptions.

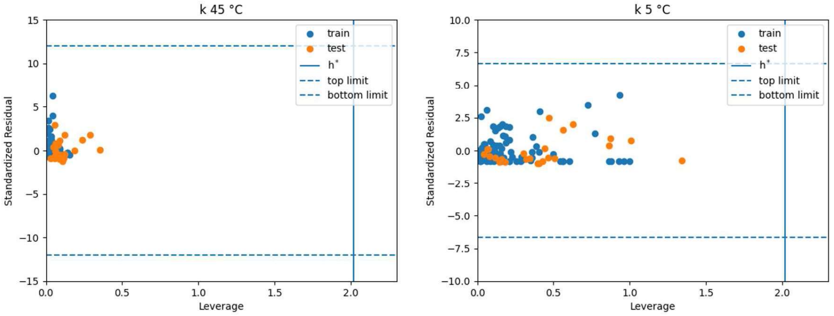

Fig. 14 Williams plots for the applicability domain of both datasets used in this work (k at 45 °C and k at 5°C).

Table 5: Feature importance at 45°C.

MD	Importance
ALOGP	0.053812087
ALOGP2	0.050708131
TDB08s	0.042568005
ESOL	0.011594299
R8s+	0.010761503
Mor21v	0.010196673
MLOGP	0.009886925
MLOGP2	0.009794333
SpMin2_Bh(s)	0.00946318
P_VSA_i_3	0.008898091
LOGP99	0.008015049
Mor14m	0.007630266
CATS3D_06_AL	0.007491519
CATS3D_07_AL	0.007223172
Mor21u	0.006953154
QED	0.006876467
CATS2D_08_AL	0.006627837

Continued on next page

Table 5: Feature importance at 45°C. (Continued)

MD	Importance
TDB10s	0.006042573
CATS3D_08_AL	0.005706398
Chi_Dz(Z)	0.005673827
QEDu	0.005546706
SpMin1_Bh(m)	0.005332613
VE3sign_B(s)	0.0050144
CATS3D_08_DL	0.004484263
SHED_NL	0.004090946
Mor11m	0.003928413
CATS2D_00_LL	0.003840408
Mor25u	0.003810378
CATS3D_05_LL	0.003632816
O-057	0.003412534
TDB09m	0.003315549
Mor12u	0.003145565
Mor23u	0.003126588
Mor20u	0.003055625
RGyr	0.002761311
SPP	0.002757774
Inflammat-80	0.002510638
qnmax	0.002399453
Mor21m	0.002354033
Mor32s	0.002286642
arLevel2	0.002276173
B09_C-O_	0.002253924

Continued on next page

Table 5: Feature importance at 45°C. (Continued)

MD	Importance
nCsp2	0.002200751
SpMin2_Bh(m)	0.002160581
minsOH	0.002157711
Mor25s	0.00209861
Depressant-80	0.002080988
P_VSA_LogP_4	0.00206184
Mor30m	0.002052736
B08_C-O_	0.002017377

Table 6: Feature importance at 5°C.

MD	Importance
CATS2D_06_NL	0.005332456
CATS2D_01_DN	0.005301684
CATS3D_06_NL	0.005184253
CATS2D_02_NL	0.005155765
CATS3D_07_NL	0.004585229
SHED_NL	0.004128087
P_VSA_ppp_N	0.003852585
Mor02s	0.003747406
NaaaC	0.003623287
nRCOOH	0.003300485
s4_numAroBonds	0.003124126
Mor23u	0.003063044
Mor23p	0.003031389

Continued on next page

Table 6: Feature importance at 5°C. (Continued)

MD	Importance
HOMT	0.002848973
SaaaC	0.002727548
TDB10m	0.002710932
O-057	0.002659734
CATS3D_08_NL	0.002583251
Infective-50	0.002544322
Mor20u	0.002523825
SHED_AN	0.002492245
arLevel1	0.00244881
TDB07v	0.002425197
CATS2D_07_AL	0.002407654
piPC08	0.002399381
CATS3D_07_AL	0.002354846
TDB09m	0.002275434
CATS2D_08_AN	0.002271158
Mor23v	0.002232561
SpMin2_Bh(s)	0.002219218
TDB09v	0.002214634
CATS2D_03_NL	0.002178151
SHED_DN	0.002171322
Mor23s	0.002157892
ALOGP	0.002157507
nCbH	0.00212561
SpMin1_Bh(m)	0.002116332
CATS2D_09_NL	0.002082708

Continued on next page

Table 6: Feature importance at 5°C. (Continued)

MD	Importance
nCb-	0.002079343
SpMax2_Bh(v)	0.002062527
CATS3D_05_NL	0.002016379
C-025	0.002008217
CATS2D_01_NL	0.001998539
CATS3D_04_NL	0.001975406
H1s	0.00197303
Mor26v	0.001970295
nBnz	0.001958582
nLevel8	0.001958556
CATS2D_02_DA	0.001956893
ALOGP2	0.001952296
GATS7p	0.001938884
TDB10s	0.001910156
Mor25v	0.001889955
R4e+	0.001883712
piPC09	0.001871591
Hypnotic-80	0.001861585
B09_O-O_	0.001833083
CATS2D_08_NL	0.001825091
Mor06u	0.001822491
arLevel2	0.001795662
ESOL	0.001756901
CATS2D_07_NL	0.001753083
mindssC	0.001752218

Continued on next page

Table 6: Feature importance at 5°C. (Continued)

MD	Importance
R1s	0.001741047
TDB10v	0.001729927
nAA	0.001726029
GATS7i	0.001725667
Uc	0.001712035
MLOGP2	0.001703991
CATS2D_04_NL	0.001689125
QED	0.001671068
TDB07m	0.001656173
RDF070m	0.001651845
piPC07	0.001635872
MDEC-23	0.001635358
RGyr	0.001634152
B08_C-C_	0.001628948
LOC	0.001598226
LOGP99	0.001591575
Mor25u	0.001587192
R2v	0.001577803
SM03_EA(dm)	0.001577518
CATS2D_00_LL	0.001575715
piPC06	0.001558089
CATS2D_05_NL	0.001557478
QEDu	0.001556226
CMC-50	0.001550571
WHALES10_Isol	0.001549674

Continued on next page

Table 6: Feature importance at 5°C. (Continued)

MD	Importance
Mor02v	0.001547303
SM12_AEA(bo)	0.001536463
G3u	0.001528736
VE3sign_D	0.001520641
s3_phRelSize	0.00151824
Hypnotic-50	0.001498807
CATS2D_06_AN	0.001483259
R8u	0.001469525
nCsp2	0.001466819
MLOGP	0.00146424
TDB08m	0.001441619
B08_C-O_	0.001423658

Following is an explanation of the descriptors used. MDs that belong to the same category or describe similar molecular properties are grouped.

- **LogP-related**: logP is a measure of the relative solubility of a compound in octanol and water, and it reflects the lipophilicity or hydrophobicity of a compound. Different methods have been developed to estimate logP from the molecular structure, using various types of atomic or molecular features. ALOGP and ALOGP2 are logP estimations based on the method of Ghose and Crippen, which considers the contribution of every atom in a molecule, including hydrogens. ALOGP2 is the square of ALOGP, which is used to account for the non-linear relationship between logP and the interphase transfer of molecules between octanol and water because too low or too high lipophilicity values act as limiting factors [40, 41]. LOGP99 is another logP

estimation based on the method of Wildman and Crippen, which uses fragment-based contributions and correction factors for hydrogen bonding and molecular size [41]. MLOGP is a calculation of logP based on 13 structural parameters, as described by Moriguchi [42]. MLOGP2 is MLOGP squared. ESOL is the estimated aqueous solubility of a molecule, which is strongly affected by logP together with the MW, the proportion of heavy atoms in aromatic systems and the number of rotatable bonds [43].

- **P_VSA type**: P_VSA_LogP_4 is a descriptor that measures the amount of molecular surface area (VSA) with a certain range of logP values. VSA is calculated by summing up the surface areas of each atom in a molecule that is not overlapped by other atoms. The surface area of each atom is determined by its van der Waals radius. The logP values are based on the Ghose-Crippen method. P_VSA_LogP_4 is one of the bins that divide the VSA based on logP values. The number 4 indicates that this bin corresponds to the fourth range of logP values [44]. P_VSA_m_3 is calculated in the same way as the P_VSA_LogP_4, except that they use a different property to weight the VSA. P_VSA_m_3 uses the mass of each atom. These descriptors are also divided into bins based on different ranges of weighted VSA values. The number 3 indicates the bin numbers. P_VSA_MR_2 is again a VSA descriptor, weighted by the molar refractivity of each atom, that is a measure of the polarizability of a molecule. In this case, the bin range is 2. P_VSA_ppp_N like other P_VSA descriptors is based on the contribution of a property to the VSA surface, the property in this case is the presence of potential negative pharmacophore points. Potential negative pharmacophore points are the regions of a molecule that have high electron density and can act as nucleophiles or hydrogen bond acceptors.
- **MoRSE**: MoRSE (Molecular Representation of Structures based on electronic diffraction) family of descriptors are based on the scattering function of electrons in a molecule, which depends on a scattering parameter, the interatomic distances,

and the number and type of atoms. The letter at the end is the weighting factor used to calculate the descriptor, and the number is the scattering parameter (the number 0 represents the first scattering parameter in this notation). The letter v uses the van der Waals volume as a weighting factor for each atom, m the mass, s the I-state, p the polarizability and u stands for unweighted. The I-state calculates the elctrotopological states of the atoms in a molecule. These latter ones are a quantification of the electronic and topological environment of the atom considered [45]. When the descriptor's value is high, the molecule has many bonds of different lengths and types.

- **Charge**: qnmax is the only descriptor from this category. It measures the maximum negative atomic charge of a molecule. It is calculated by using quantum chemical methods that assign partial charges to each atom in a molecule [46].
- **Burden matrix**: The Burden matrix is a modified connectivity matrix representation of molecular structure that uses atomic masses as diagonal elements and bond orders as off-diagonal elements. VE3sign_B(s) It is a spectral descriptor that is derived from the eigenvectors of the Burden matrix that corresponds to the direction of the matrix. VE3sign_B(s) uses the logarithmic coefficient sum of the last eigenvector from the Burden matrix weighted by I-State [47]. While the descriptors SpMax2_Bh(s), SpMin1_Bh(m) and SpMin4_Bh(s) are respectively the largest (Max) or smallest (Min) eigenvalue for the number reported in the name, of the Burden matrix, weighted by I-state if (s) or mass if (m).
- **WHALES**: WHALES or Weighted Holistic Atom Localization and Entity Shape, are descriptors obtained from different molecular properties, such as geometric interatomic distances, molecular shape, and partial charge distribution. They are calculated from an atom-centred covariance matrix that is used to normalize the interatomic distances proportional to one of the following 3 atomic indices: remoteness (Rem), isolation degree (Iso) or isolation-remoteness (IR) [48].

- **MDE**: These descriptors are molecular distance-edge vector calculations for specific atoms in a molecule. They are based on the distance between atoms in the molecular graph and the edges of the adjacency in the graph [49]. After the word MDE there is a letter that stands for the nature of the atom and 2 numbers depicting the type of the atom, e.g., 1 is primary. In the models presented in this work, there are 2 of these descriptors MDEO-11 and MDEC-23, the first one the MDE of primary oxygens, and the latter one the MDE of secondary and tertiary carbons.
- **Moran autocorrelation**: Moran autocorrelation descriptors are a class of 2D autocorrelation descriptors that are based on the concept of molecular distance matrix. They measure the spatial autocorrelation of a molecular property (such as mass, volume, electronegativity, or polarizability) by using the Moran coefficient as a weighting factor. They are topological descriptors that capture the distribution and similarity of atoms in a molecule [10]. MATS2e measures the lag 2 autocorrelation weighted by Sanderson electronegativity [50]. Where lag refers to the topological distance between 2 atoms which are distanced by 2 edges in this case, in a molecular graph. A high absolute value of MATS2e indicates the uniform distribution of electronegativity in the molecule.
- **Broto-Moreau autocorrelation**: Broto-Moreau autocorrelation is another type of 2D autocorrelation derived from interaction geodesic matrices [10]. ATS7m corresponds to the autocorrelation index for the lag 7 weighted by mass.
- **CATS**: CATS (Chemical Advanced Template Search) family of descriptors encode the topological distances between pharmacophore points in the molecule. CATS can be calculated in 2D or 3D, depending on whether the molecular structure is represented by a graph or a set of coordinates. The numbers indicate the distance between two pharmacophoric features, either in bond lengths (for 2D) or in angstroms (for 3D). The letters indicate the type of pharmacophoric features: N for negative, P for positive, L for lipophilic, D for donor and A for acceptor [51].

- **WHIM**: WHIM (Weighted Holistic Invariant Molecular) descriptors are calculated from the PCA of a weighted covariance matrix that represents the molecular coordinates of the 3D structure of a molecule (spatial conformation), and they contain information about its size, shape, symmetry, and atom distribution. They use different weighting schemes: u for unweighted, m atom mass, v for van der Waals volume, e Mulliken atomic electronegativity, p atomic polarizability, s electro topological indices of Kier and Hall, scaled for the carbon atom, and i for ionization potential [52]. E1m is the only MD appearing from this category. It is calculated by using the first principal component of the covariance matrix weighted by mass.
- **Balaban indices**: J_G/D is the only descriptor that belongs to this category. Balaban indices are calculated from a reciprocal distance symmetric matrix of the molecule that represents the topological distances of its atoms (count of the edges in the shortest path between two vertexes) [53]. G/D indicates that the matrix used is the geometrical distance matrix.
- **Hyper-Wiener-like indices**: This case also only presents one descriptor amongst our features, HyWi_G. Such descriptors are derived from a matrix whose elements are Wiener's numbers (W). The number W is defined as the sum of the distance between any two carbons in the molecule in terms of C-C bonds [54].
- **2D atom pairs**: All the descriptors from this category that appear in our features are B type, meaning that they represent the presence or absence of a certain fragment of atoms expressed as a frequency at a specific distance [55]. The number expresses the topological distance and the letter between underscores is the fragment, e.g., _C-O_ stands for carbon-oxygen.
- **SHED**: SHED descriptors use the Shannon entropy concept to quantify the variability in the distribution of pharmacophoric feature pairs in a molecule. Shannon entropy is a measure of the uncertainty or randomness of a system. A higher entropy means a more diverse or complex system. They are calculated from the

topology of the molecule, using the shortest paths between feature pairs [56]. The pharmacophoric feature letters are the same as for CATS.

- **Functional groups count**: nRCOOH is simply the number of aliphatic carboxylic acids present in the molecule [10].
- **Ring descriptors**: nBnz is the number of benzene-like rings [10].
- **Constitutional indices**: nCsp2 is the number of sp2 hybridized carbon atom [10].
- **TDB**: TDBs are topological autocorrelation descriptors based on the sum of a property weighted by a function of the Euclidean distance between pairs of atoms in a molecule [57, 58]. The number represents the distance considered while the letter is the weight used for the calculation (s is the I-state, m is the mass, and v is the van der Waals volume).
- **Path counts**: A piPC descriptor or molecular multiple path counts is the path count weighted by the bond order. The path count is the number of unique paths of length k [10]. The number is the bond order.
- **E-state**: E-state indices are related to the electron topological state of the molecules. NaaaC in this context, is the number of aaCa atom groups, where the a stands for aromatic, C is carbon, and SaaaC is the sum of E-states of the carbons that belong to the same group, hence, they are surrounded by three aromatic groups. The mindssC corresponds to the minimum E-state of carbons with one double bond and two single bonds [45].
- **Chirality**: A few descriptors related to chirality are also present. The arLevel1 and 2 describe the number of neighbouring aromatic atoms of the chiral centre at levels 1 and 2 respectively, nLevel8 is the number of neighbouring atoms of the chiral centre at level 8 and s4_numAroBonds, the number of aromatic bonds of the substituent 4 [10].

- **Radical Centric Information Index**: ICR is the number of graph vertices having the same atom eccentricity, that is, the maximum distance from a vertex to any other vertex in the molecular graph [10].
- **GETAWAY**: GEometry, Topology, and Atom-Weights AssemblY (GATEWAY) are 3D molecular descriptors based on a leverage matrix calculated from the spatial coordinates of the molecule atoms weighted by chemical information. There are two types of GATEWAY descriptors, H-type which is derived using only the influence of the matrix and R-type which combines the influence of the matrix with the influence of a geometry matrix. Thanks to the combination of the two matrices, R-type descriptors contain useful information related to the presence of significant substituents or fragments in the molecule, they reflect the atomic properties distribution together with the leverages over geometric distance of atom pairs in the molecule along specific topological distances. R8u is R-autocorrelation of lag 8 unweighted. The R4e+, is the maximal R-autocorrelation of lag 4 weighted by electronegativity, referring to a pair of atoms having the highest electronegativity and leverage at a topological distance of 4 in the molecule. The H-type is further divided into 2 types, HATS and H. HATS describes the atomic properties distribution together with the leverage information of the atoms, and H describes the distribution together with the accessibility degree between the atoms in the molecule along specific topological distances [59]. Where again the number corresponds to the topological distance space and the letter is the weight, with the same notation as previous descriptors.
- **Geary autocorrelation**: Geary autocorrelation coefficients represent the distribution of a certain property within specific topological distances, describing the structure homogeneity. Low values mean strong autocorrelation. Positive autocorrelation translates in values between 0 and 1 whereas negative autocorrelation produces values larger than 1 [60]. Next to the acronym GATS, the numbers and

letters assume the same meaning as previously described descriptors (space and weight).

- **Atom-centred fragments**: The MDs O-057 described the presence of oxygen in specific substructures, phenol, enol, and carboxyl OH [10].

- **CoMMA**: QYYm is calculated through comparative molecular moment analysis (CoMMA). This method uses moments of the molecular mass and charge distribution to build the MDs. Amongst the MDs calculated in this way, there are two that relate solely to molecular charge, the magnitude of the dipole moment p, and the magnitude of the principal quadrupole moment Q. Q can be divided into two quadrupolar components, QXX and QYY because it is calculated for a translated inertial reference frame [61]. QYYm is the quadrupole Y-component weighted by the mass.

  Drug-like: Three MDs describe the drug-likeness that belongs to two categories. The first one is QEDu (quantitative estimate of drug-likeness unweighted). It is calculated averaging by the geometric mean of 8 functions that represent each different parameter. The 8 parameters are molecular mas, octanol-water partition coefficient, number of hydrogen bond donors, number of hydrogen bond acceptors, molecular polar surface area, number of rotatable bonds, number of aromatic rings and number of structural alerts. Overall, QEDu quantifies the compound desirability, its values range from 0, when all the properties are unfavourable, to 1, when all the properties are favourable [62]. The other 2 MDs that describe drug-likeness are calculated from the Ghose-Viswanadhan-Wendoloski quantitative and qualitative characterization of drug-like compounds based on the CMC (comprehensive medicinal chemistry) database. They also consider similar properties as the previous descriptor, molecular weight, number of atoms, logP, and molar refractivity, and they are determined in two ranges, a qualifying range that covers 80% of the compounds and a preferred

range that is 50% of the compounds [63]. CMC-50 simply refers to this drug-like index at 50%, and Infective-50 is based on the antiinfective-like index at 50%.

- **RDF**: Radial distribution functions (RDF) are the mathematical representation of the 3D structure of a molecule derived from the infrared spectroscopy monitored vibration of the atoms in the molecule. It can be interpreted as the probability distribution to find an atom in a spherical volume. These MDs can also be weighted by a property, in our case, RDF060v is weighted by the van der Waals volume. They are encoded in 30 discrete values corresponding to the distance from a reference point in a range of $1 - 15.5$ $\AA$ with a step of 0.5 $\AA$, the number indicates the range [64].
